# The effects of transcranial magnetic stimulation for freezing of gait in Parkinson’s disease: a systematic review and meta-analysis of randomized controlled trials

**DOI:** 10.3389/fnagi.2024.1304852

**Published:** 2024-02-02

**Authors:** Zicai Liu, Xin Wen, Xiuying Xie, Yangyou Liu, Cheng Tan, Shuanghong Kuang, Huiyu Liu

**Affiliations:** ^1^Department of Rehabilitation Medicine, Shaoguan First People’s Hospital, Shaoguan, Guangdong, China; ^2^Yuebei People’s Hospital, Shaoguan, Guangdong, China

**Keywords:** transcranial magnetic stimulation, freezing of gait, Parkinson’s disease, meta-analysis, TMS

## Abstract

**Background:**

Freezing of gait (FOG) is one of the most disabling gait disturbances in Parkinson’s disease (PD), affecting mobility and balance severely, thereby leading to an increased risk of falls.

**Objectives:**

The purpose of this systematic review and meta-analysis was to investigate the effects of transcranial magnetic stimulation on FOG in PD.

**Methods:**

Based on PRISMA guidelines, we searched the databases of MEDLINE (PubMed), Cochrane Library, PEDro, Embase, and Web of Science. Studies of the English language published up to July 2023 were searched. We retrieved for studies of randomized controlled trials (RCTs) of transcranial magnetic stimulation to treat FOG after PD and screened by inclusion and exclusion criteria. Risk of bias was assessed using the Cochrane Collaboration’s tool (Revman5.30). Characteristics of RCTs were extracted. The heterogeneity of the trials was measured by *I^2^* statistic. The effect size was expressed by a standardized mean difference (SMD) with a 95% confidence interval (CI).

**Results:**

A total of 488 articles were screened, after screening sixteen RCTs involved in 408 patients were included in the qualitative analysis, and 15 RCTs were included in meta-analysis. The outcome measures included FOG-Q, walking time, TUG, and UPDRS. Six studies used FOG-Q as outcome measure, six studies used walking time, four studies used TUG, and six studies used UPDRS. Compared with placebo treatment, transcranial magnetic stimulation has positive significant effects in improving gait status with increased walking speed (SMD = −0.41, 95% CI = −0.75 to −0.06, *I^2^* = 7% *p* = 0.02), FOG-Q scores (SMD = −0.55, 95% CI = −0.89 to −0.21, *I^2^* = 29%, *p* = 0.002), UPDRS scores (SMD = −1.08, 95% CI = −1.39 to −0.78, *I^2^* = 49%, *P* < 0.001) and the time of TUG (SMD = −0.56, 95% CI = −0.88 to −0.23, *I^2^* = 25%, *p* = 0.02) decreased.

**Conclusion:**

Transcranial magnetic stimulation could significantly improving gait conditions in PD patients with FOG.

**Systematic review registration:**

https://www.crd.york.ac.uk/PROSPERO/#recordDetails, CRD42023434286.

## Introduction

Parkinson’s disease (PD) is a progressive neurodegenerative illness of the central nervous system that usually affects middle-aged and elderly people (Frisardi et al., 2016), and is reported to increase in prevalence with ages (Hirsch et al., 2016). The main neural mechanism of PD is decreased in dopamine levels in the basal nucleus, namely the dorsal striatum. Epidemiological studies have shown that the prevalence of PD is 1 to 2% in persons 65 years of age or older (Weintraub et al., 2008), 4% in persons 80–89 years of age (Noyes et al., 2006). Parkinson’s disease is clinically characterized by non-motor symptoms such as mood and affective disorders and sleep disorders, as well as motor symptoms such as resting tremor, bradykinesia, rigidity, postural instability and gait disturbances (Bloem et al., 2021). Freezing of gait (FOG) is one of the most disabling gait impairments in PD. A study included 990 patients with PD presented that the incidence of FOG was 32% (Giladi et al., 1992). FOG is an episodic phenomenon defined as a brief, episodic absence or marked reduction of forward progression of the feet despite the intention to walk (Nutt et al., 2011). FOG usually occurs in situations where the gait is erratic, such as when turning a corner or going through a narrow passageway (Snijders et al., 2008). FOG seriously affects mobility, leads to increased risk of fall (Latt et al., 2009; Kerr et al., 2010) and poor quality of life (Moore et al., 2007; Rahman et al., 2008).

The most common treatment for motor symptoms in PD is dopamine-based pharmacologic treatments, and other treatments such as deep brain stimulation (Xie et al., 2017) and magnetic resonance imaging-guided focused ultrasound (Xu et al., 2021) have also been used. In addition, a variety of exercise interventions may improve motor symptoms to varying degrees (Gilat et al., 2021). The efficacy of pharmacologic treatments decreases over time, and adverse effects become apparent and other treatments’ therapeutic effect is limited to some extent. The treatment of FOG is difficult, and despite the optimal pharmacologic and nonpharmacologic interventions are used, the majority of patients will still develop FOG (Bloem et al., 2015).

Transcranial magnetic stimulation (TMS) is a valuable non-invasive neuromodulation technique for modulating brain activity in a specific, distributed, cortico-subcortical network (Fregni and Pascual-Leone, 2007). High frequency TMS (≥5 Hz) could enhance motor cortex excitability (Gilio et al., 2002), whereas low frequency TMS (≤1 Hz) could downregulate cortical excitability (Chen et al., 1997). In recent years, TMS has been shown to be as a potential treatment for improving motor signs in PD (Elahi et al., 2009; Chou et al., 2015; Zhu et al., 2015; Chung and Mak, 2016). Some previous studies have demonstrated that TMS has a beneficial effect on FOG in PD (Xie et al., 2020; Deng et al., 2022). However, the studies referenced in the previous systematic reviews included crossover studies in addition to randomized control studies (RCTs), and the limited number of RCTs failed to provide sufficient evidence.

Therefore, we aimed to conduct a systematic review and meta-analysis of the RCTs assessing the efficacy of TMS on FOG in PD to offer an evidence-based basis for clinical treatment. Previous meta-analyses included studies that were not all RCTs; in recent years, studies have been updated, and our systematic review will only include all RCTs to improve the quality of evidence from our study.

## Materials and methods

### Protocol and registration

This systematic review was designed and conducted according to the Preferred Reporting Items for Systematic Reviews and Meta-analysis (PRISMA) guideline (Page et al., 2021). The study has been registered with Prospero (registration number: CRD42023434286).

### Search strategy

Five large databases which included MEDLINE (PubMed), Cochrane Library, PEDro, Embase, and Web of Science were searched from inception through July 2023. In the process of searching for studies, we only considered studies in English. The studies were retrieved by using the keywords “Parkinson’s disease” OR “disease of Parkinson” OR “Parkinson disorders” AND “freezing of gait” OR “gait disturbances” OR “gait” AND “transcranial magnetic stimulation” OR “TMS.” Furthermore, we also manually retrieved for studies that appeared in other systematic reviews that might be related to our study.

### Eligibility criteria

The studies were included if they met the PICOS criteria as follows: (1) population (P): all of patients were adults older than 18 years diagnosed with freezing of gait in PD; (2) intervention (I): TMS; (3) control (C): placebo stimulation or no intervention was considered as the control; (4) outcomes (O): freezing of gait questionnaire (FOG-Q) as primary outcome and walking time, Unified Parkinson’s Disease Rating Scale (UPDRS) and Time up and Go (TUG) as secondary outcomes; and (5) study design (S): RCTs.

The exclusion criteria were as follows: (1) duplicate studies, (2) case-controlled trials, (3) full article was not available, and (4) fail to extract the valid outcome data.

### Study selection and data acquisition

Firstly, the retrieved studies were imported into the EndnoteX20 document management system, and repeated records were deleted. Secondly, two reviewers (LZC and WX) independently screened the title and abstract of the identified studies and excluded those that were not relevant. The full texts of the potentially relevant studies were further reviewed strictly according to the predesigned eligibility inclusion. Afterwards, we confirmed the final included studies after reviewing the full text. The inconsistencies of study selection were settled by discussion with another reviewer (LHY).

Two investigators (LZC and XXY) independently extracted the following information from each included study: subject characteristics, treatment methods, outcome measures, treatment duration, main parameters of TMS using a standardized extraction form. Discrepancies of data extraction were resolved by discussion with another researcher (TC).

### Quality assessment

The quality of the included randomized controlled studies was assessed by two authors (WX and LYY) independently using the Cochrane Collaboration’s tool (Revman5.30) (Jørgensen et al., 2016). Risk bias assessment contains seven aspects: random sequence generation, allocation concealment, blinding of participants and personnel, blinding of outcome assessment, incomplete outcome data, selective reporting, and other bias. Disagreements were resolved by intragroup discussion or with another experienced researcher (LHY).

### Statistical analysis

All statistical analyses were performed using the Review Manager (RevMan 5.3) software. A Chi square test evaluated the statistical significance of heterogeneity. The *I^2^* was reported as a measure of heterogeneity, *I*^2^ > 50% was interpreted as substantial heterogeneity, the random-effect model was applied to describe the center of the distribution of intervention effects. For quantitative synthesis, pooled-effect estimates were obtained by comparing the changes from baseline to the post-intervention across groups or directly comparing the post-intervention scores of each group. The effect size was expressed by a standardized mean difference (SMD) with a 95% confidence interval (CI).

## Results

### Literature search

**Figure 1:**
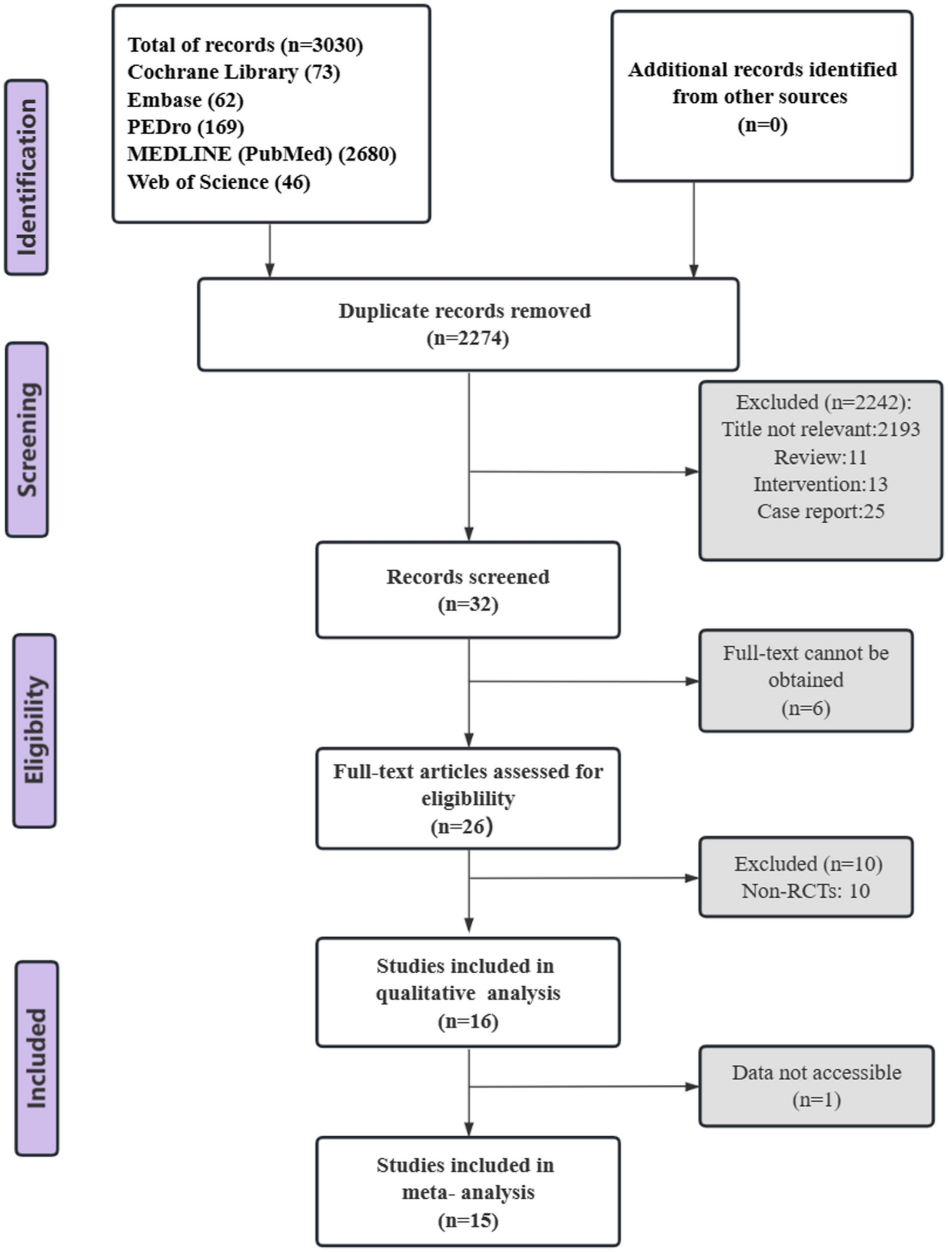
PRISMA flow diagram.

We initially searched 3,030 records from five electronic databases. After deleting duplicates, screening the titles and abstracts, 36 records remained for further assessment. After reviewing the full text, we excluded 16 articles for the following reasons: and full-text not available (*n* = 6) and non-RCTs (*n* = 10). Eventually, 16 RCTs were included in the qualitative analysis and 15 RCTs in meta-analysis. Figure 1 summarized the inclusion process.

### Study characteristics

Sixteen RCTs with a total of 408 patients were included in our systematic review. The included studies were published between 2003 and 2021, and the sample size of the included studies ranged from 13 to 50. The average age of the patients ranged from 54.3 to 71.6, and the average duration of disease ranged from 3.5 to 13.8 years. A study (Benninger et al., 2011) used intermittent theta-burst stimulation (iTBS), a study used deep TMS, and the remaining studies used conventional rTMS as the intervention. The treatment time of TMS ranged from 1 session to 24 sessions. The outcome measures included FOG-Q, walking time, TUG, and UPDRS. Six studies (Benninger et al., 2012; El-Tamawy et al., 2013; Lee et al., 2014; Ma et al., 2019; Mi et al., 2019; Lench et al., 2021) used FOG-Q as outcome measure, six (Khedr et al., 2003, 2006; Lomarev et al., 2006; del Olmo et al., 2007; Benninger et al., 2011, 2012) studies used walking time, four studies (Yang et al., 2013; Lee et al., 2014; Cohen et al., 2018; Lench et al., 2021) used TUG, and six studies used UPDRS (Khedr et al., 2003; Arias et al., 2010; Benninger et al., 2012; Chung et al., 2020; Li et al., 2020; Lench et al., 2021). The detailed characteristics of the included studies were summarized in Tables 1, 2 (Khedr et al., 2003, 2006; Lomarev et al., 2006; del Olmo et al., 2007; Benninger et al., 2011, 2012; El-Tamawy et al., 2013; Yang et al., 2013; Lee et al., 2014; Jørgensen et al., 2016; Cohen et al., 2018; Ma et al., 2019; Mi et al., 2019; Li et al., 2020; Lench et al., 2021; Page et al., 2021).

**Table 1:** Characteristics of participants included in studies.

Study	Patients (M/F)	Age (years)	Disease duration (years)	H&Y stage	Interventions
Khedr et al. (2003) Egypt	EG: 19 (14/5) CG: 17 (10/7)	EG: 57.8 ± 9.2 CG: 57.5 ± 8.4	EG: 3.45 ± 2.3 CG: 3.05 ± 2.1	2–3	EG: real-rTMS CG: sham-rTMS
Lomarev et al. (2006) USA	EG: 9 (7/2) CG: 9 (8/1)	EG: 63 ± 10 CG: 66 ± 10	EG: 13.8 ± 6.8 CG: 10.8 ± 3.1	2–4	EG: real-rTMS CG: sham-rTMS
Khedr et al. (2006) Egypt	EG: 10 CG: 10	EG: 60.2 ± 9.48 CG:60.6 ± 10.6	EG: 3.5 ± 0.7 CG: 3.8 ± 0.9	3–5	EG: real-rTMS CG: Occupational stimulation
del Olmo et al. (2007) Spain	EG: 8 CG: 5	61.7 ± 5.22	8.0 ± 5.0	1–3	EG: real-rTMS CG: sham-rTMS
Arias et al. (2010) Spain	EG: 9 CG: 9	Not reported	Not reported	2–4	EG: real-rTMS CG: sham-rTMS
Benninger et al. (2011) Switzerland	EG: 13 (7/6) CG: 13 (11/2)	EG: 62.1 ± 6.9 CG: 65.6 ± 9.0	EG: 10.8 ± 7.1 CG: 6.5 ± 3.4	EG: 2.6 ± 0.2 CG: 2.5 ± 0.1	EG: real-iTBS CG: sham-iTBS
Benninger et al. (2012) Switzerland	EG: 13 (11/2) CG: 13 (9/4)	EG: 55.8 ± 9.1 CG:54.3 ± 12.5	EG: 8.6 ± 4.1 CG: 9.3 ± 6.8	EG: 2.7 ± 0.3 CG: 2.9 ± 0.6	EG: real-rTMS CG: sham-rTMS
El-Tamawy et al. (2013) Egypt	16 (11/5)	67.0 ± 7.32	Not reported	3.1 ± 0.6	EG: real-rTMS CG: sham-rTMS
Yang et al. (2013) China	EG: 10 (5/5) CG: 10 (7/3)	EG: 65.2 ± 11.1 CG: 67.0 ± 13.2	EG: 6.4 ± 2.7 CG: 6.4 ± 3.6	EG: 2.3 ± 0.4 CG: 2.4 ± 0.4	EG: real-rTMS CG: sham-rTMS
Lee et al. (2014) Korea	20 (13/7)	71.6 ± 8.6	4.7 ± 2.6	3.4 ± 0.5	EG: real-rTMS CG: sham-rTMS
Cohen et al. (2018) Israel	EG: 21 (17/4) CG: 21 (15/6)	EG: 66.4 ± 4.8 CG: 66.8 ± 8.1	EG: 4.7 ± 3.4 CG: 5.6 ± 3.7	2–4	EG: real-rTMS CG: sham-rTMS
Ma et al. (2019) China	EG: 18 (8/10) CG: 10 (5/5)	EG: 59.9 ± 9.2 CG: 66.0 ± 8.6	EG: 8.9 ± 5.5 CG: 7.5 ± 4.7	Not reported	EG: real-rTMS CG: sham-rTMS
Mi et al. (2019) China	EG: 20 (9/11) CG: 10 (5/5)	EG: 62.7 ± 10.6 CG: 65.6 ± 8.7	EG: 9.2 ± 5.8 CG: 7.4 ± 4.8	EG: 2.6 ± 0.9 CG: 2.4 ± 0.9	EG: real-rTMS CG: sham-rTMS
Chung et al. (2020) China	EG1: 17 (10/7) EG2: 17 (9/8) CG: 16 (7/9)	EG1:62.7 ± 6.8 EG2: 62.1 ± 5.7 CG: 62.1 ± 5.7	EG1: 5.2 ± 3.4 EG2: 7.5 ± 4.9 CG: 6.9 ± 3.3	EG1: 2.2 ± 0.3 EG2: 2.2 ± 0.4 CG: 2.3 ± 0.3	EG1: 25 Hz-rTMS EG2: 1 Hz-rTMS CG: sham-rTMS
Li et al. (2020) China	EG: 24 (16/8) CG: 24 (16/8)	EG: 61.7 ± 6.9 CG: 61.5 ± 8.4	EG: 5.5 ± 3.7 CG: 6.5 ± 5.1	EG: 1.9 ± 0.6 CG: 1.8 ± 0.6	EG: real-rTMS CG: sham-rTMS
Lench et al. (2021) USA	EG:12 (7/5) CG: 8 (7/1)	EG: 66.6 ± 7.5 CG: 64.5 ± 8.9	EG: 8.7 ± 7.1 CG: 8.0 ± 5.6	EG: 2.3 ± 0.4 CG: 2.3 ± 0.3	EG: real-rTMS CG: sham-rTMS

USA, the United States of America; EG, experimental Croup; CG, control Croup; M, male; F, female; rTMS, repetitive transcranial magnetic stimulation; iTBS, intermittent theta burst stimulation.

**Table 2:** Main parameters of TMS.

Study	Coil type	rTMS site	Frequency	Intensity	No. of pulse *session	Trains	Treatment duration	Post-evaluation	Outcomes
Khedr et al. (2003)	F8	M1 + DLPFC	5 Hz	120%MT	2000*10	Not reported	10 days	10 days; Post_1m_	Time of the 25-m walk; motor section of the UPDRS
Lomarev et al. (2006)	F8	M1 + DLPFC	25 Hz	100%MT	1200*8	Not reported	4 weeks	4 weeks; Post_1m_	Time of the 10-m walk
Khedr et al. (2006)	F8	M1	10 Hz	100%MT	3000*6	20 tps of 5 s	6 days	6 days	Time of the 25-m walk
del Olmo et al. (2007)	F8	DLPFC	10 Hz	90%RMT	450*10	15 tps of 1 s	10 days	1 day	Walking time
Arias et al. (2010)	C	M1	1 Hz	90%RMT	600*10	50 tps	10 days	10 days	Motor section of the UPDRS
Benninger et al. (2011)	C	M1 + DLPFC	iTBS (50 Hz)	80%AMT	600*8	20 tps of 2 s	2 weeks	2 weeks; Post_1m_	Time of the 10-m walk
Benninger et al. (2012)	C	M1	50 Hz	80%AMT	1000*8	Not reported	2 weeks	2 weeks; Post_1m_	Time of the 10-m walk; FOG-Q; motor section of the UPDRS III
El-Tamawy et al. (2013)	F8	M1	1 Hz	90%MT	500*12	10 tps of 50 s	4 weeks	4 weeks	FOG-Q
Yang et al. (2013)	F8	M1	5 Hz	100%RMT	1200*12	24 tps of 10 s	4 weeks	4 weeks	TUG
Lee et al. (2014)	double-cone; F8	M1; SMA; DLPFC	10 Hz	90%RMT	1000*1	20 tps of 5 s	1 day	1 days	FOG-Q; TUG
Cohen et al. (2018)	H-coil	M1 + PFC	1/10 Hz	110/100%MT	900/800*24	40 tps of 2 s	3 months	3 months	TUG
Ma et al. (2019)	F8	SMA	10 Hz	90%RMT	1000*10	20 tps of 5 s	10 days	12 days, Post_1m_	FOG-Q
Mi et al. (2019)	F8	SMA	10 Hz	90%RMT	1000*10	20 tps of 5 s	10 days	12 days, Post_1m_	FOG-Q
Chung et al. (2020)	double-cone	bilateral M1	1 Hz, 25 Hz	80%RMT	1200*12	Not reported	3 weeks	1 day, Post_1m_	TUG; MDS- motor section of the UPDRS III
Li et al. (2020)	F8	M1	20 Hz	80%RMT	2000*5	20 tps of 5 s	1 week	1 week	MDS- motor section of the UPDRS III
Lench et al. (2021)	F8	SMA	1 Hz	110%RMT	1200*10	Not reported	10 days	10 days	FOG-Q; motor section of the UPDRS-III

F8, figure-eight coil; M1, primary motor cortex; DLPFC, dorsolateral prefrontal cortex; MT, motor threshold; Post_1m_, 1 month postintervention; tps, trains per session; UPDRS: Unified Parkinson’s Disease Rating Scale; rTMS, repetitive transcranial magnetic stimulation; RMT, resting motor threshold; C, circular; AMT, active motor threshold; FOG-Q, freezing of gait questionnaire; TUG, time up and go; SMA, supplementary motor area; PFC, prefrontal cortex; MDS-UPDRS III, Movement Disorder Society–Unified Parkinson’s Disease Rating Scale motor score Part III.

### Result of quality assessment

**Figure 2:**
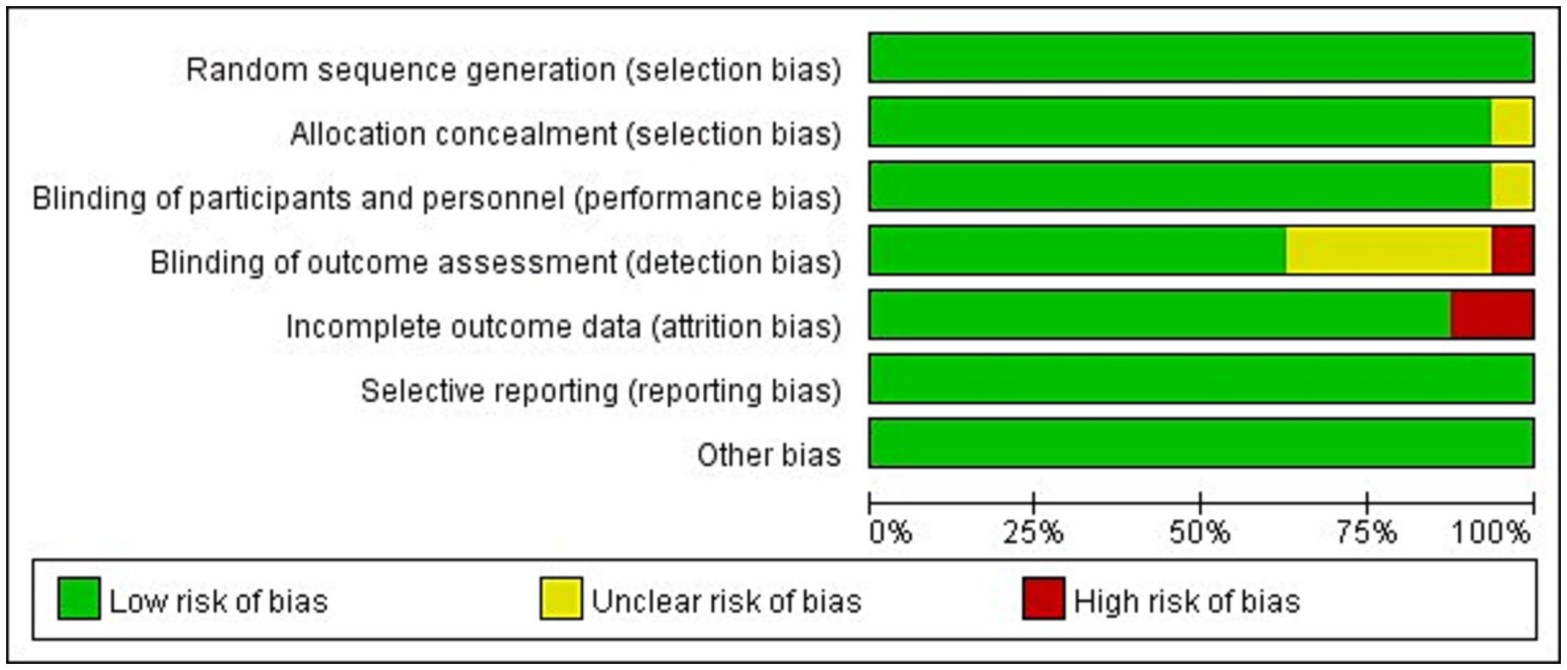
Risk of bias items shown as percentages across the included studies.

**Figure 3:**
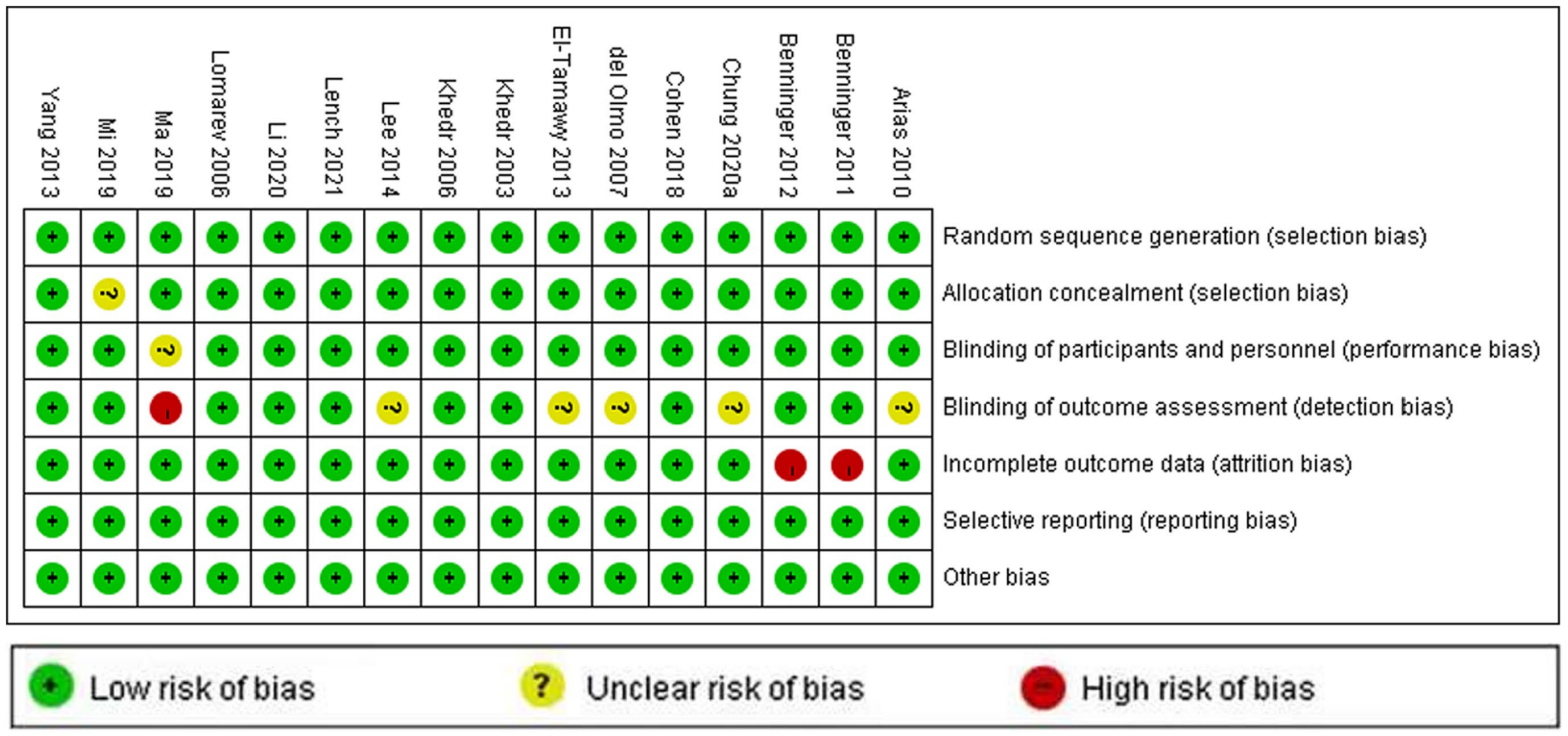
Risk of bias assessment for 16 RCTs.

The methodological quality and bias risk assessment according to the Cochrane risk of bias tool (Revman5.30) for included study were presented in Figures 2, 3. In general, the risk of bias for the included studies was relatively low.

### Results of analysis

**Figure 4:**
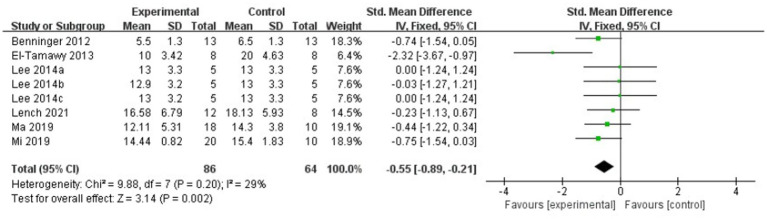
Forest plot for FOG-Q comparison, standard mean difference.

**Figure 5:**
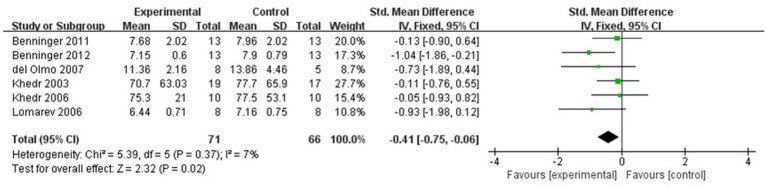
Forest plot for walking time comparison, standard mean difference.

**Figure 6:**
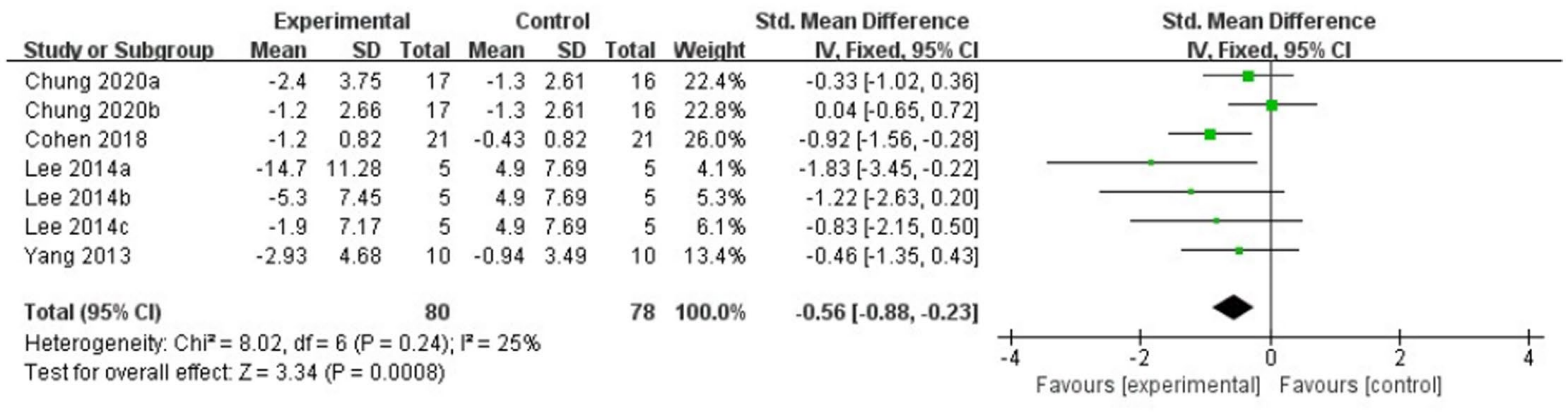
Forest plot for TUG comparison, standard mean difference.

**Figure 7:**
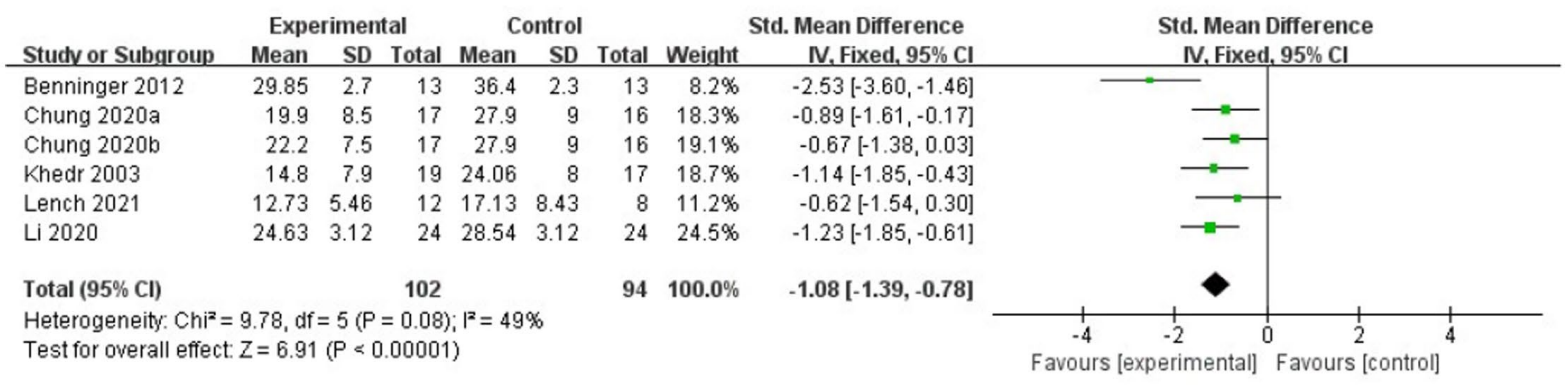
Forest plot for UPDRS comparison, standard mean difference.

The meta-analysis results showed that the FOG-Q scores for the rTMS group were better than those for the control group (fixed effects model, SMD = −0.55, 95% CI = −0.89 to −0.21, *I^2^* = 29%, *p* = 0.002, Figure 4). Besides, our study found that real rTMS treatment had a significantly beneficial effects on accelerating walking speed (expressed in walking time) compared to placebo-controlled treatment (fixed effects model, SMD = −0.41, 95% CI = −0.75 to −0.06, *p* = 0.02) with low heterogeneity (*I^2^* = 7%, Figure 5). Four studies evaluated TUG (expressed in walking time) and were included in the quantitative analysis. rTMS had a significantly beneficial effect on the mean changes in the difference from baseline to post-intervention compared with sham stimulation (fixed effects model, SMD = −0.56, 95% CI = −0.88 to −0.23, *I^2^* = 25%, *p* = 0.02, Figure 6). In addition, Figure 7 represented the post-intervention effects of rTMS for the scores of UPDRS, our meta-analysis indicated that rTMS had significantly beneficial post-intervention effects compare with control group (fixed effects model, SMD = −1.08, 95% CI = −1.39 to −0.78, *I^2^* = 49%, *p* < 0.001, Figure 7).

**Figure 8:**
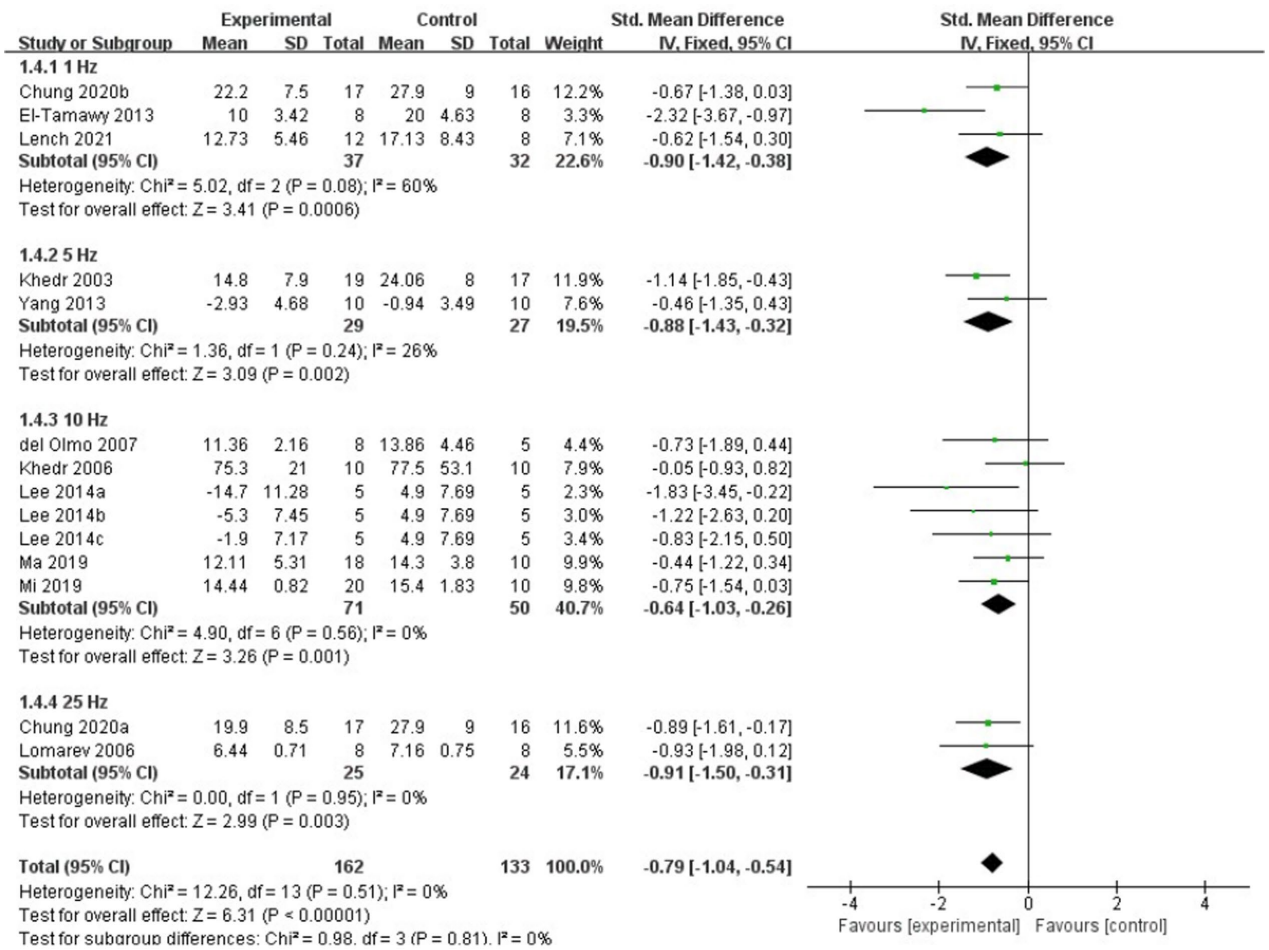
Forest plot for subgroup analysis according to the stimulation frequency.

**Figure 9:**
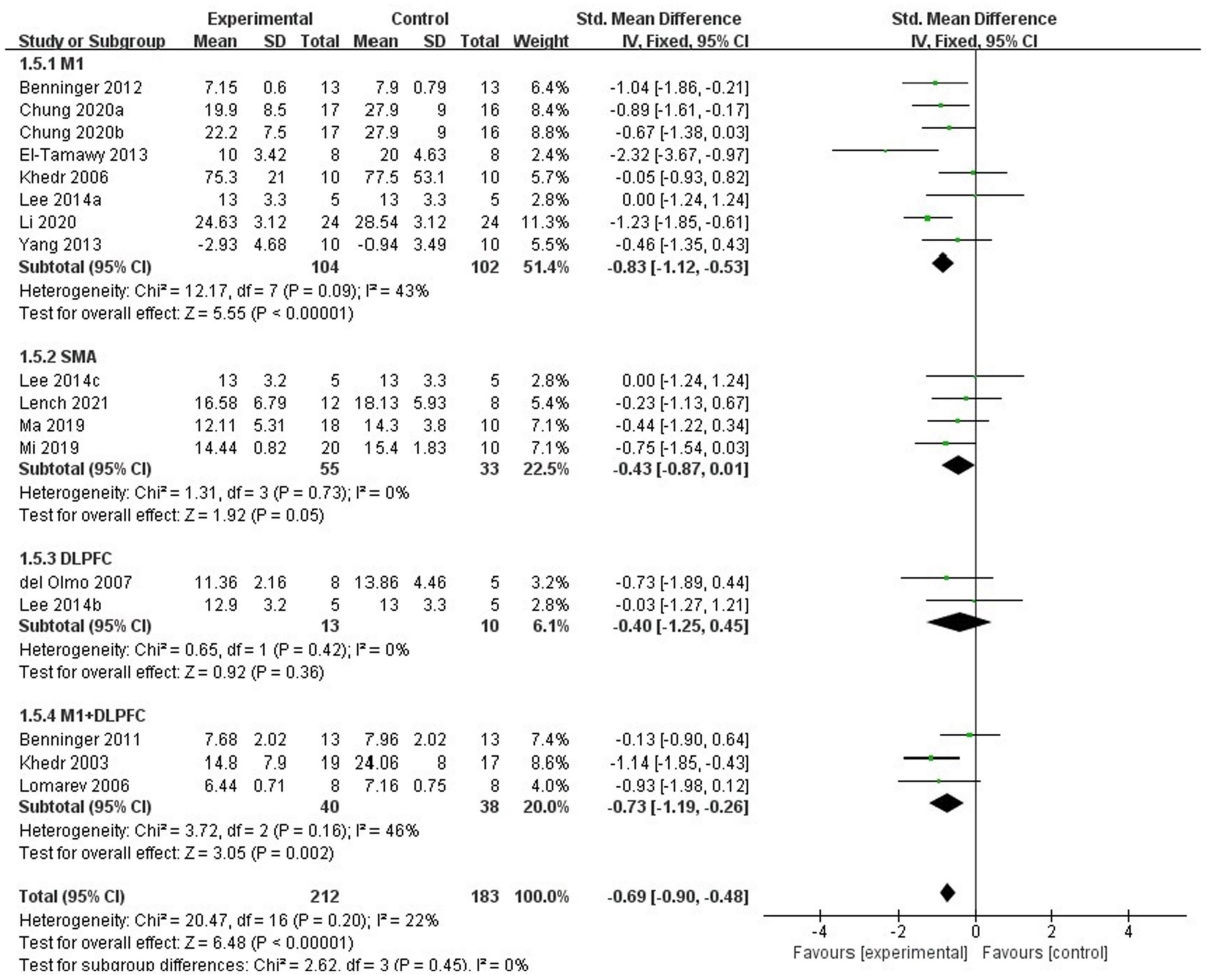
Forest plot for subgroup analysis according to the stimulation site.

For subgroup analysis, TMS treatment of 1 Hz, 5 Hz, 10 Hz, and 25 Hz have significant efficacy (SMD = −0.79, 95% CI = −1.04 to −0.54, Figure 8), and the stimulation frequency of 25 Hz had a better effect size than the other frequencies (SMD = −0.91, 95% CI = −1.50 to −0.31, Figure 8). However, no statistically significant difference was found between different stimulation frequency (*I^2^* = 0%, *p* = 0.81, Figure 8). The result of subgroup analysis based on stimulation site showed that the effect size of primary motor cortex (M1) was the largest (SMD = −0.83, 95% CI = −1.12 to −0.53, Figure 9), and TMS of the dorsolateral prefrontal cortex (DLPFC) has no significant effect on FOG in PD patients (fixed effects model, SMD = −0.40, 95% CI = −1.25 to −0.45, *I^2^* = 0%, *p* = 0.36, Figure 9). Furthermore, there was no significant difference between different subgroups based on stimulation sites (*I^2^* = 0%, *p* = 0.45, Figure 9).

## Discussion

The purpose of our study was attended to investigate the effectiveness of TMS combined or not with other treatments on FOG after PD. In this systematic review and meta-analysis, we reviewed 16 RCTs of TMS in patients with FOG after PD. Our study discovered that real TMS intervention was more effective than placebo treatment for improvement of gait condition with accelerated walking speed after a period of intervention. This result was consistent with that of previous studies (Xie et al., 2020; Deng et al., 2022).

Overall, TMS is a relatively safe treatment. Out of 16 studies, only six reported adverse effects in patients during stimulation (Khedr et al., 2006; Benninger et al., 2011; El-Tamawy et al., 2013; Lee et al., 2014; Cohen et al., 2018; Li et al., 2020). Frequent adverse reactions are headache, dizziness, nausea, local pain and discomfort, which are mostly transient. Furthermore, no studies have reported serious adverse effects during stimulation. A study by Rossi et al. (2021) reported that induction of seizures is the most serious adverse effect of TMS, whereas risk of TMS to induce seizures is certainly very low. Similarly, seizures were not reported in any of the sixteen studies included in our study. The stimulation parameters of transcranial magnetism may be an important factor affecting safety. The stimulus intensity applied in the study was at 80–110% RMT, and this range of stimulus intensity is also considered to be safer. Flitman et al. (1998) reported an episode of a generalized tonic clonic seizure in a healthy subject using parameters of 120% of MT, 15 Hz, train duration of 0.75 s, and with variable intervals between trials. This may be due to intervals that are too short or the intensity of the stimulus. To ensure safety, consider reducing the stimulation duration and increasing the intervals when the stimulation frequency and intensity are high.

The result of our meta-analysis based on frequency demonstrated that TMS of 1 Hz, 5 Hz, 10 Hz, and 25 Hz had significant effects in improving gait status when compared to the sham stimulation. Furthermore, subgroup analyses also revealed that TMS of 25 Hz has a greater effect size in comparison to other frequencies. Chung et al. (2020) compared the effects of 1 Hz, 25 Hz and sham stimulation on gait and motor performance. It was found that 1 or 25 Hz TMS prior to treadmill training enhanced and prolonged the effects of training on gait and motor performance compared to sham stimulation. However no significant treatment difference was found between 1 Hz and 25 Hz stimulation. In addition to this, there are no other studies directly comparing the effects of different frequencies of TMS on FOG in patients with PD. A randomized, double-blinded, cross-over study by Kim et al. (2015) reported that 10 Hz rTMS over the M1 area of the dominant hemisphere for 5 sessions in a week has significant improvements. A study by Benninger et al. (2012) concluded that 50 Hz rTMS over theM1 area could not improve motor performance and functional status in patients with PD. As far as current study is concerned, there is no agreement on the optimal stimulation frequency for TMS for FOG in patients with PD.

The results also showed that TMS of DLPFC had no significant benefit in improving gait status when compared to the sham stimulation group. The results of our study are similar to those of del Olmo et al. and different from those of Lee et al. A study by del Olmo et al. (2007) indicated that rTMS of the DLPFC not have a significant benefit on the performance of motor tasks in PD patients. Lee et al. (2014) demonstrated that there was a positive effect of 10 Hz rTMS over the DLPFC on FOG and gait function. Dagan et al. (2017) reported that deep rTMS over the middle prefrontal cortex (mPFC) could not impact the severity of FOG. Additionally, the result of subgroup analysis showed greater effect sizes for TMS therapy over M1, SMA, or M1 combined with DLPFC compared to placebo treatment. However, there were no significant differences between stimulation of the M1 vs. the SMA vs. M1 combined with DEPLFC. Seven studies (Khedr et al., 2006; Arias et al., 2010; Benninger et al., 2012; El-Tamawy et al., 2013; Yang et al., 2013; Chung et al., 2020; Li et al., 2020) have targeted the M1 region for TMS stimulation. A study by El-Tamawy et al. (2013) though rTMS over the M1 may have a therapeutic effect in on-freezers with advanced PD. Chung et al. (2020) found that 1 and 25 Hz rTMS groups produced a greater improvement in fastest walking speed at post-intervention than the sham group. These studies also reported that TMS over the M1 have positive effects on the improvement of gait performance (Khedr et al., 2006; Arias et al., 2010; Maruo et al., 2013; Yang et al., 2013; Li et al., 2020). A fMRI study noted that 25 Hz rTMS to the bilateral M1 increased functional connectivity between the SMA and prefrontal areas during complex motor tasks (González-García et al., 2011). However, Benninger et al. (2012) proposed that 50 Hz rTMS of the M1 did not improve gait in PD. Fricke et al. (2019) hypothesized that TMS may activate subpopulations of neurons in the hypothalamic nucleus through direct projections from cortical neurons to different cortical areas (e.g., primary motor cortex), and that abnormal amplitude activity in the hypothalamic nucleus may be associated with motor symptoms in PD. The abnormal amplitude activity of the hypothalamic nucleus may be related to the motor symptoms of PD, and TMS stimulation may be able to change these abnormal amplitudes. They hypothesized that persistent decoupling of hypothalamic nucleus neurons could be improved by combined two-site TMS. They performed combined two-site TMS on dorsal premotor cortex and primary motor cortex in 20 patients with PD, and the results showed that combined two-site TMS had no clinically meaningful beneficial effects on motor symptoms in PD.

Three studies (Ma et al., 2019; Mi et al., 2019; Lench et al., 2021) have targeted the SMA area for TMS stimulation. The results of these studies all found that rTMS over the SMA could improve the gait performances in patients with PD, which was consistent with our findings. The SMA is located anterior to the M1 leg area, and the SMA is important in several types of motor processes and is activated before movement initiation (Nachev et al., 2008). Therefore, Kim et al. suggest that SMA stimulation is a more-appropriate target in PD patients with FOG (Kim et al., 2018). Three studies (Khedr et al., 2003; Lomarev et al., 2006; Benninger et al., 2011) have targeted the M1 combined with DLPFC regions for TMS stimulation. The findings of these previous studies indicated that rTMS has a positive effect on improvement in motor performance in FOG after PD, which was in agreement with our results. A study by Lee et al. (2014) noted rTMS over the M1, SMA, and DLPFC all induced greater effects than placebo treatment and rTMS over the M1, DLPFC have a greater effect compare to SMA, but no significant differences were found between the M1 and the DLPFC stimulation. However, Kim et al. (2018) believed that SMA is a more appropriate target than the MI area for brain stimulation when treating PD patients with FOG, which was not consistent with the results of Lee et al. Another randomized cross-over pilot study compared the effects of 10 Hz rTMS over the MI and DLPFC on the patients with FOG after PD, they concluded that no significant effect of rTMS over the DLPFC and M1 on FOG, but has a trend toward improvement of the Stroop test interference after rTMS over the DLPFC (Rektorova et al., 2007). In addition, a previous study also suggested that theta burst stimulation (TBS) over the cerebellar does not improve FOG in patients with PD (Janssen et al., 2017). As far as current research is concerned, there is no agreement on the optimal brain stimulation target for TMS for FOG in patients with PD.

However, there are several limitations to this study. First of all, the studies included in quantitative analysis were dissimilar regarding the severity of symptoms, disease duration, and the time of TMS therapies. In addition, the sample size of these studies was relatively small. Therefore, the final results should be carefully interpreted. Furthermore, the risk of bias in some areas was not clear due to incomplete data in a few studies, which limited the results.

## Conclusion

TMS therapy presented some significant benefits on improvement of gait and motor performance. However, the results of subgroup analyses based on different frequencies and different brain stimulation targets did not show significant differences. Further large studies are required in the future to investigate the optimal stimulation parameters for TMS in patients with FOG in PD. Although it has been reported that TMS may cause side effects such as headache, dizziness, nausea, and malaise, these adverse stimuli are mostly transient, and TMS can be considered a relatively safe treatment. In conclusion, TMS had a positive and significant effect in improving gait such as increased walking speed, FOG-Q score, UPDRS score and reduced TUG time compared to placebo treatment. This suggests that transcranial magnetism is an effective treatment modality for FOG in PD.

## Data availability statement

The original contributions presented in the study are included in the article/supplementary material, further inquiries can be directed to the corresponding authors.

## Author contributions

ZL: Conceptualization, Writing – original draft, Writing – review & editing, Data curation, Resources. XW: Investigation, Methodology, Software, Writing – original draft. XX: Data curation, Formal analysis, Project administration, Writing – original draft. YL: Data curation, Formal analysis, Supervision, Writing – original draft. CT: Project administration, Resources, Supervision, Validation, Writing – original draft. SK: Writing – review & editing, Writing – original draft, Funding acquisition. HL: Investigation, Project administration, Resources, Supervision, Validation, Visualization, Writing – original draft.
